# Chemical Exchange Saturation Transfer MRI Signal Loss of the Substantia Nigra as an Imaging Biomarker to Evaluate the Diagnosis and Severity of Parkinson's Disease

**DOI:** 10.3389/fnins.2017.00489

**Published:** 2017-08-31

**Authors:** Chunmei Li, Min Chen, Xuna Zhao, Rui Wang, Haibo Chen, Wen Su, Shuhua Li, Baohui Lou, Guodong Song, Shuai Zhang, Jintao Zhang, Jinyuan Zhou

**Affiliations:** ^1^Department of Radiology, Beijing Hospital, National Center of Gerontology Beijing, China; ^2^Department of Radiology, Johns Hopkins University Baltimore, MD, United States; ^3^Department of Neurology, Beijing Hospital, National Center of Gerontology Beijing, China

**Keywords:** CEST imaging, APT imaging, biomarker, substantia nigra, Parkinson's disease

## Abstract

The early diagnosis of Parkinson's disease (PD) and the accurate evaluation of disease severity are crucial for intervention and treatment in PD patients. In this study, we applied chemical exchange saturation transfer (CEST) imaging to patients at different stages of PD and explored the clinical value of the CEST signal loss of the substantia nigra as an imaging biomarker of PD. The measured CEST signal intensities (including amide proton transfer-weighted or APTw, and total CEST or CEST_total_) of the substantia nigra in PD patients showed a significantly decreased tendency with PD progression. Compared to normal controls, the APTw and CEST_*total*_ intensities of PD patients significantly decreased at both the early and advanced or late stages. These APTw and CEST_total_ values of the substantia nigra were also significantly lower in advanced or late stage PD patients than in early stage PD patients. For PD patients with unilateral symptoms, the APTw and CEST_total_ values in the substantia nigra on the affected side were significantly lower than those in normal controls. Both the APTw and CEST_total_ values of PD were significantly correlated with the severity of disease and disease duration. Our findings suggest that the CEST MRI signal of the substantia nigra is a potential imaging biomarker for the diagnosis and monitoring of the severity of PD.

## Introduction

Parkinson's disease (PD) is a common progressive neurodegenerative disease characterized by the loss of dopaminergic neurons in the substantia nigra (Fearnley and Lees, 1991). Currently, the diagnosis of PD is mainly based on the clinical manifestations. Therefore, the accuracy of the diagnosis is often unsatisfactory, even for experienced movement-disorder specialists (Hughes et al., 1992; Tolosa et al., 2006). The situation is even worse for patients at the early stage of PD, when the clinical manifestations can be atypical. However, the early detection of PD would make early intervention possible, which may greatly improve the prognosis for PD patients. In addition to the diagnosis, monitoring disease severity, which plays an important role in disease-modifying therapy, is also crucial for PD patients. Consequently, it is essential to find a novel, reliable method for PD diagnosis and severity evaluation.

Previous studies have indicated that the dopaminergic neuron loss in the substantia nigra has a strong correlation with the progressive clinical manifestations of PD (Ma et al., 1997), which makes substantia nigra imaging a promising biomarker for the diagnosis and severity evaluation in PD patients. With the rapid development of medical imaging techniques, various novel MR techniques have been used in the PD research focused on the substantia nigra, including diffusion tensor imaging (Kim et al., 2013; Aquino et al., 2014; Du et al., 2014), susceptibility-weighted imaging (Cosottini et al., 2014; He et al., 2015), functional MR imaging (Ellmore et al., 2013; Manza et al., 2016), and MR spectroscopy (Zhou et al., 2014; Seraji-Bozorgzad et al., 2015). However, in clinic no reliable *in vivo* MR biomarkers have been found, as yet, for PD diagnosis and severity evaluation.

Recently, a new class of MRI contrast, termed chemical exchange saturation transfer (CEST) (Ward et al., 2000), has been proposed. CEST imaging is a new molecular MRI method that allows detection of low-concentration chemicals with exchangeable protons through the water signal (Zhou and Van Zijl, 2006; Sherry and Woods, 2008). Based on the CEST approach, Zhou et al. have proposed a technique called amide proton transfer (APT) imaging (Zhou et al., 2003), which can generate image contrast based on endogenous cellular proteins and peptides in tissue. In recent years, different groups have performed a number of clinical investigations, ranging from neoplastic disorders [in the brain (Zhou et al., 2013b; Togao et al., 2014; Jiang et al., 2016), prostate (Jia et al., 2011), breast (Dula et al., 2013), and neck (Yuan et al., 2014)] to non-oncologic diseases [such as cerebral ischemia (Sun et al., 2007; Tietze et al., 2014; Harston et al., 2015), epilepsy (Davis et al., 2015), and pediatric brain development and delay (Zhang et al., 2016)]. In our recent work, it was revealed that the CEST/APT signals in the substantia nigra were decreased (Li et al., 2014), especially for the advanced stage of PD. Although we found the potential of CEST/APT in the detection and classification of PD patients firstly, there are still some issues need further investigation, such as the correlation between CEST/APT and PD severity.

The aim of this study was to assess the potential of using the CEST/APT signal in the substantia nigra as a biomarker in the diagnosis and evaluation of PD progression. We classified patients according to the H&Y scale and the disease duration, and analyzed the CEST/APT signal intensity changes of PD patients at different stages. We also assessed the CEST/APT signal intensity in the bilateral substantia nigra of PD patients with unilateral symptoms. In addition, we evaluated the correlations between CEST/APT signal intensity in the substantia nigra and the severity of PD, quantified by several different clinical scales.

## Materials and methods

### Subjects

Sixty-one PD patients (34 men and 27 women; mean age 63 years) and 24 normal controls (13 men and 11 women; mean age 65 years) were enrolled in this study. For the PD group, an experienced movement-disorders specialist (15 years of experience) made the diagnosis of PD according to published criteria (Hughes et al., 1992, 2001). All the patients fulfilled the United Kingdom Parkinson's Disease Society brain bank diagnostic criteria. They were evaluated with the H&Y scale, the unified Parkinson's disease rating scale (UPDRS), the Mini-Mental State Examination (MMSE), the Hamilton Depression Scale (HAMD), and the Hamilton Anxiety Scale (HAMA) by the same movement-disorder specialist, while their medications were stopped for at least 12 h.

The exclusion criteria for this study were: a history of head trauma; central nervous system infection; exposure to anti-dopaminergic drugs; other neurologic or psychiatric diseases; or a structural lesion or hydrocephalus on brain magnetic resonance images. This study was approved by the Institutional Review Board of Beijing Hospital (registration number: 2015BJYYEC-006-01). Written, informed consent was obtained from each subject.

### MRI protocol

MR imaging was performed on a 3 Tesla MRI system (Achieva 3T; Philips Medical Systems, Best, The Netherlands), using an eight-channel sensitivity-encoding coil for reception. Pencil beam second-order shimming was employed. For the PD patients, MR images were acquired shortly after the neurological assessment. For both the PD patients and normal controls, routine images, including axial T_2_-weighted, T_1_-weighted, and fluid-attenuated inversion recovery (FLAIR), were first obtained to confirm that there was no structural abnormality.

The CEST/APT imaging sequence was based on a pseudo-continuous wave, off-resonance RF irradiation and a single-shot, turbo-spin-echo readout. The parameters were as follows: repetition time = 3,000 ms; turbo-spin-echo factor = 54; field of view = 230 × 221 mm; matrix = 105 × 100 (reconstructed to be 400 × 400); slice thickness = 6 mm. A transverse slice that included the midbrain was acquired. The slice with substantia nigra on FLAIR images was picked out and CEST/APT image of the same slice would be acquired. A pseudo-continuous wave RF irradiation (saturation duration = 200 ms × 4; inter-pulse delay, 10 ms; power level = 2 μT) and a multi-offset, multi-acquisition CEST/APT imaging protocol (Li et al., 2014) were used. The 31 offsets were 0, ±0.25, ±0.5, ±0.75, ±1 (2), ±1.5 (2), ±2 (2), ±2.5 (2), ±3 (2), ±3.25 (2), ±3.5 (8), ±3.75 (2), ±4 (2), ±4.5, ±5, ±6 ppm (the values in parentheses represented the number of acquisitions, which was 1, if not specified). For signal normalization, an unsaturated image was obtained. The acquisition time of the CEST/APT image scan was about 3 min.

### Data processing

The Interactive Data Language (IDL, ITT Visual Information Solutions, Boulder, CO, USA) was used for image analysis. The measured magnetization transfer spectra were corrected for B_0_ field inhomogeneity effects on a pixel-by-pixel basis. CEST/APT imaging was calculated by the magnetization transfer ratio (MTR = 1-S_sat_/S_0_) asymmetry (MTR_asym_) analysis with respect to the water resonance (S_sat_ and S_0_ represent the signal intensities with and without selective RF irradiation, respectively, plotted as a function of saturation frequency offset, relative to water). The calculated APTw images were called APT-weighted (APTw) images (Zhou et al., 2013a; Heo et al., 2016a,b). Meanwhile, the total CEST signal intensity, CEST_total_, was defined as the integral of the MTR_asym_ spectrum in the range of 0–4 ppm.

**Figure 1:**
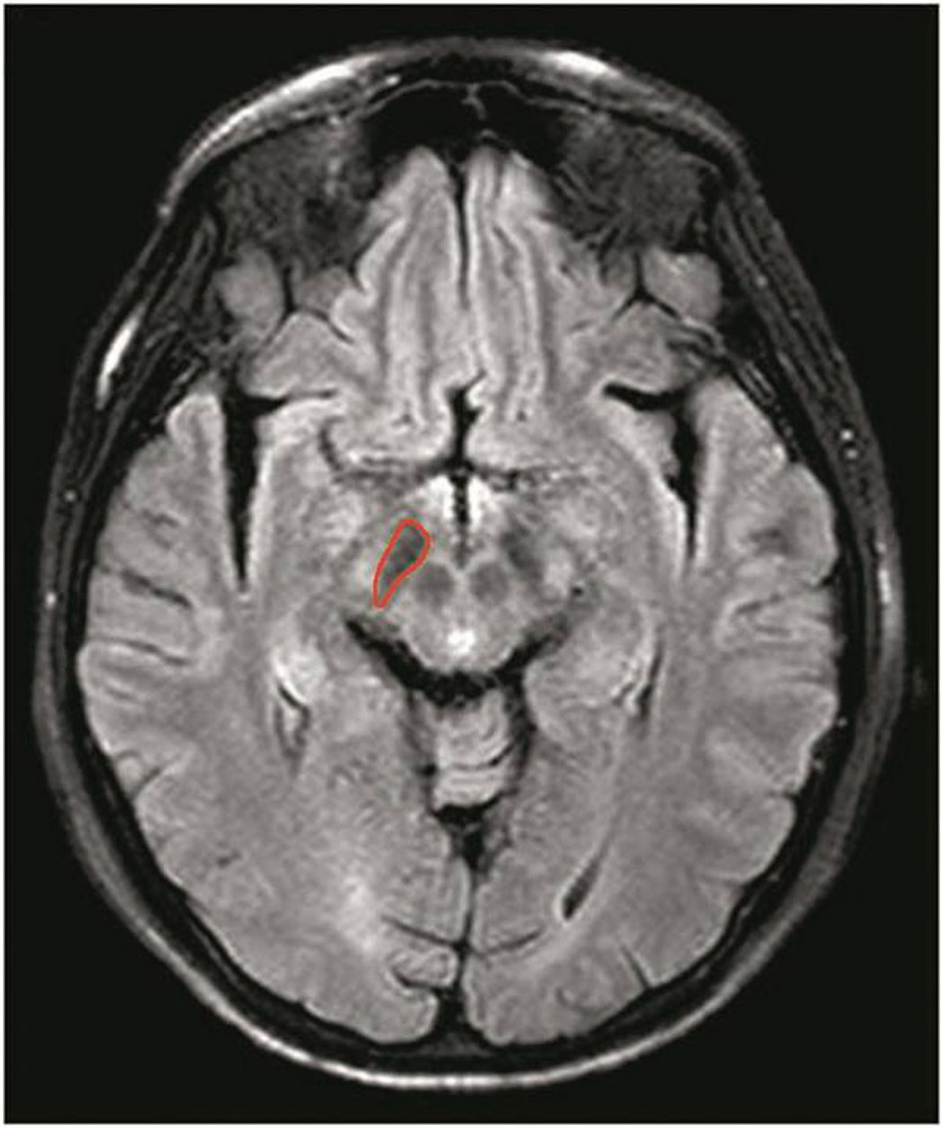
Example of the definition of the region of interest for quantitative analysis of the substantia nigra. The FLAIR images were used as the anatomical reference when drawing regions of interest on the whole substantia nigra regions on M0 map.

Two radiologists (CL and RW, who had 6 and 11 years of experience in neurological imaging, respectively) performed the quantitative image analysis for PD patients and normal controls. The FLAIR images were used as the anatomical reference to draw regions of interest on the whole substantia nigra regions on M0 map (Figure 1). Besides, we assessed the normal-appearing white matter (NAWM) of the occipital lobe in the midbrain to determine whether there existed changes in CEST signals between PD patients and normal controls.

### Statistical analysis

All data were analyzed using the statistical package SPSS16.0. Sixty-one PD patients were divided into two groups with two different classifications: (I) based on the H&Y scale—the early stage (H&Y scale ≤ 2) and the advanced stage (H&Y scale ≥ 2.5); (II) based on the disease duration—the early stage (disease duration ≤ 3 year) and the late stage (disease duration > 3 years). The age and sex differences among normal controls and two PD groups were tested by ANOVA and χ^2^ test, respectively, and there was no significance between groups (*P* > 0.05).

For PD patients with unilateral symptoms, we defined the contralateral side of symptoms as the affected side and the ipsilateral side as the unaffected side. The CEST/APT imaging intensities of the affected side were recorded as a sample in the PD group, while those of the unaffected side were not included. For patients with bilateral symptoms, the CEST/APT imaging intensities of both sides were recorded as two separate samples in the PD group. The average CEST/APT imaging intensities (APTw and CEST_total_) and corresponding 95% confidence intervals were calculated for the substantia nigra of PD patients at different stages. One-way ANOVA was used to compare the CEST signal differences among normal controls and PD patients at different stages. We further analyzed whether there were differences among the normal controls, affected side, and unaffected side with one-way ANOVA, specifically for PD patients with unilateral symptoms. Correlation analysis was performed for the CEST signal of the substantia nigra and clinical assessment, including the H&Y scale, the UPDRS, the MMSE, the HAMD, and the HAMA. For the UPDRS, correlations between the CEST signal of the substantia nigra and UPDRS-I (relating to mind, behavior, and emotion), UPDRS-II (relating to the activities of daily living), UPDRS-III (relating to motor complications), UPDRS-IV (relating to treatment complications), and UPDRS-V (relating to other complications) were also calculated. The level of significance was set at *P* < 0.05. UPDRS-II, the UPDRS-III, and the UPDRS-IV measure the activities of daily living, motor complications, and treatment complications.

## Results

### Z spectrum and MTR_asym_ spectrum

**Figure 2:**
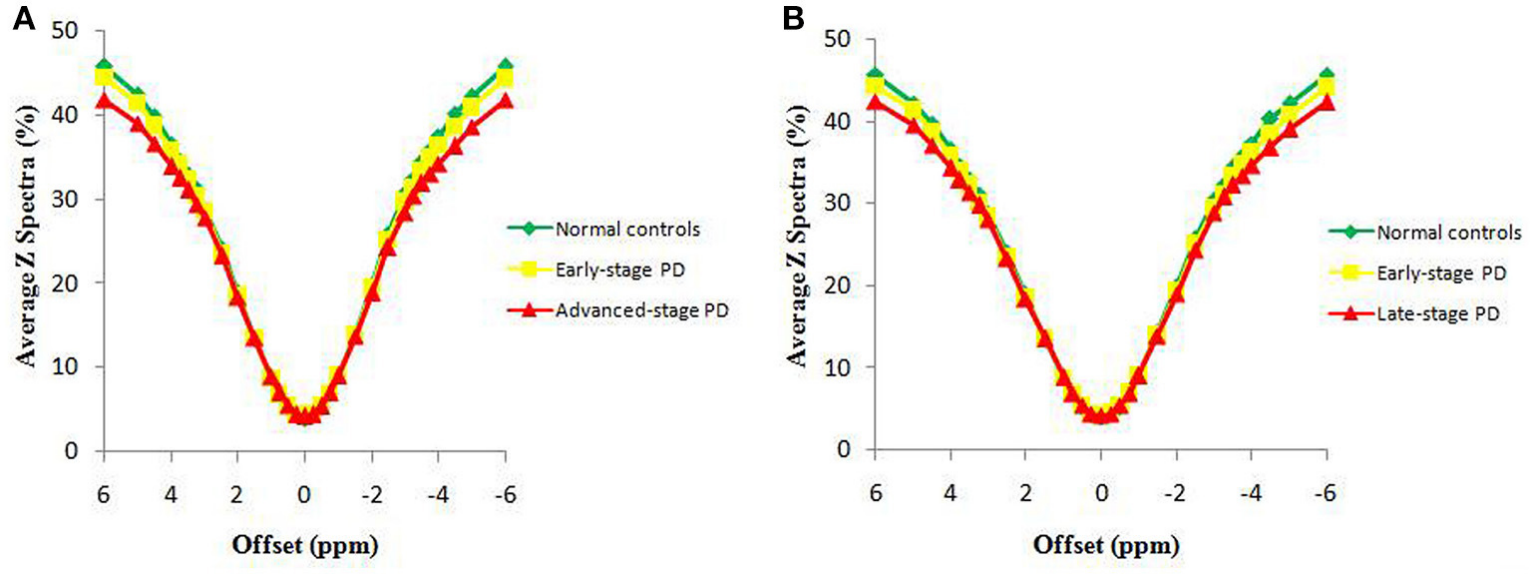
The average Z spectra of the substantia nigra for three groups, based on the classification of H&Y scale **(A)** and based on the classification of disease duration **(B)**.

**Figure 3:**
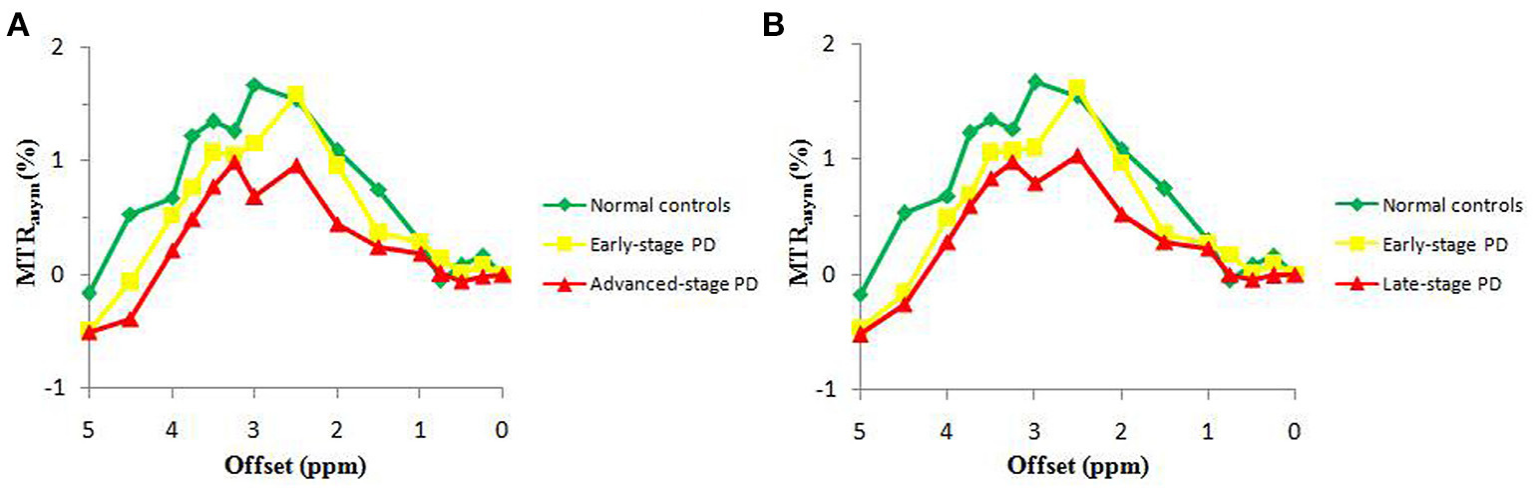
The average MTRasym spectra of the substantia nigra for three groups, based on the classification of H&Y scale **(A)** and based on the classification of disease duration **(B)**.

Figure 2 compares the average Z spectra of three groups in the substantia nigra, which were based on the classification of H&Y scale and the classification of disease duration. The average Z spectrum was highest for the normal controls and lowest for the advanced/late stage PD patients. Figure 3 compares the average MTR_asym_ spectra of three groups in the substantia nigra, which were based on the classification of H&Y scale and the classification of disease duration. The APTw value (MTR_asym_ at 3.5 ppm) was highest for the normal controls and lowest for the advanced/late stage PD patients.

### Diagnosing PD with the CEST/APT signal

**Figure 4:**
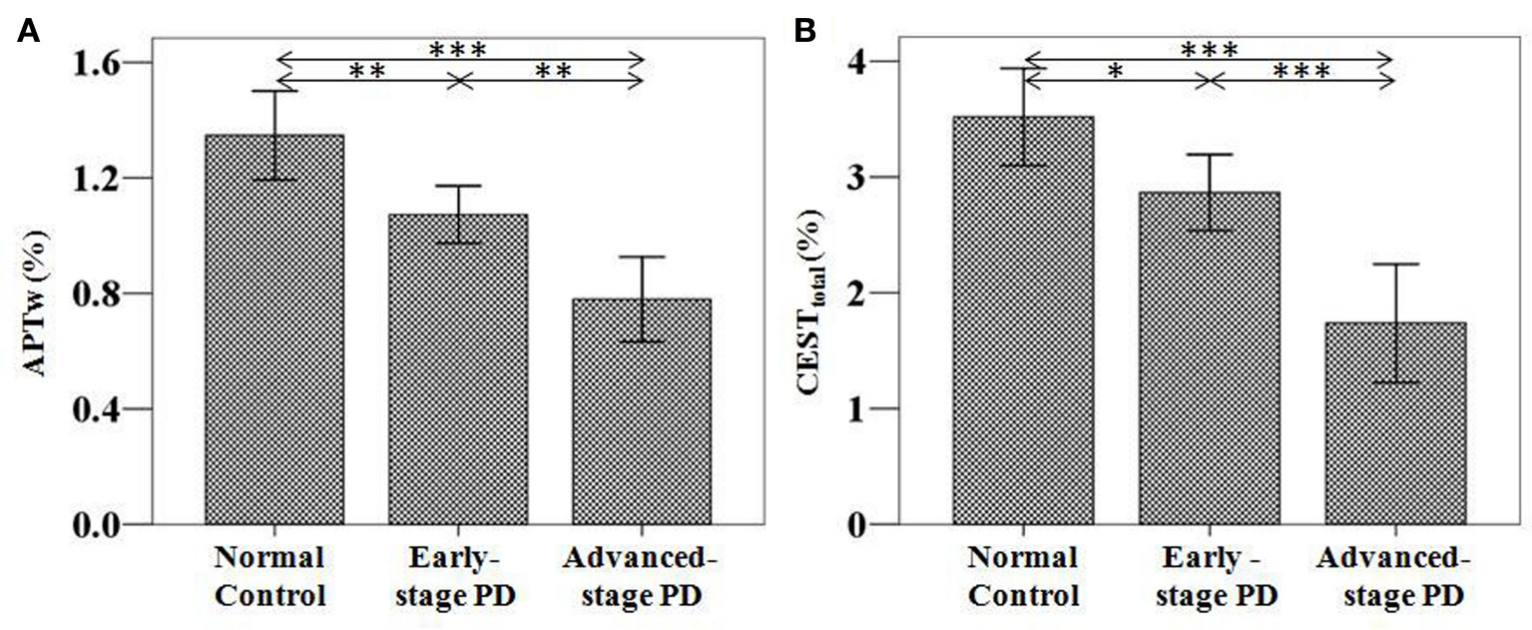
APTw **(A)** and CEST_total_ **(B)** values of the substantia nigra for normal controls (*n* = 24) and PD patients at different stages (grouped by the H&Y scale) (*n* = 38 for early-stage and *n* = 23 for advanced-stage). The APTw and CEST_total_ values of the substantia nigra for normal controls, early-stage, and advanced-stage PD patients were (1.35 ± 0.15) and (3.52 ± 0.42)%, (1.07 ± 0.10) and (2.87 ± 0.34)%, (0.78 ± 0.15) and (1.74 ± 0.52)%, respectively. Both showed a gradual decrease with disease progression. ^*^P < 0.05; ^**^P < 0.01; ^***^P < 0.001.

Based on the classification based on the H&Y scale, 55 samples from 38 patients at the early stage of PD were acquired. For the unilateral symptoms group (21 patients), we select one sample on the contralateral substantia nigra of symptoms (21 samples); for the bilateral symptoms group (17 patients), we selected two samples on the bilateral substantia nigra (34 samples). Forty-six samples from 23 patients at the advanced stage were also acquired (no patients with unilateral symptoms). The results are shown in Figure 4 and Table 1.

**Table 1:** APTw and CEST_total_ values (mean ± 95% CI) of the substantia nigra for normal controls and Parkinson's disease (PD) patients at different stages (classification according to the H&Y scale).

	**Normal (*n* = 24)**	**Early-stage PD (*n* = 38)**	**Advanced-stage PD (*n* = 23)**	***P*-values**
APTw (%)	1.35 ± 0.15	1.07 ± 0.10	0.78 ± 0.15	**0.003, 0.002**,<**0.001**
CEST_total_(%)	3.52 ± 0.42	2.87 ± 0.34	1.74 ± 0.52	**0.025**,<**0.001**,<**0.001**

*APTw and CEST_total_ were quantified by % water signal intensity. The three P-values correspond to the results of the comparison between normal controls and early stage PD patients, between early-stage and advanced-stage PD patients, and between normal controls and advanced stage PD patients, respectively. Bold indicates a significant change*.

**Figure 5:**
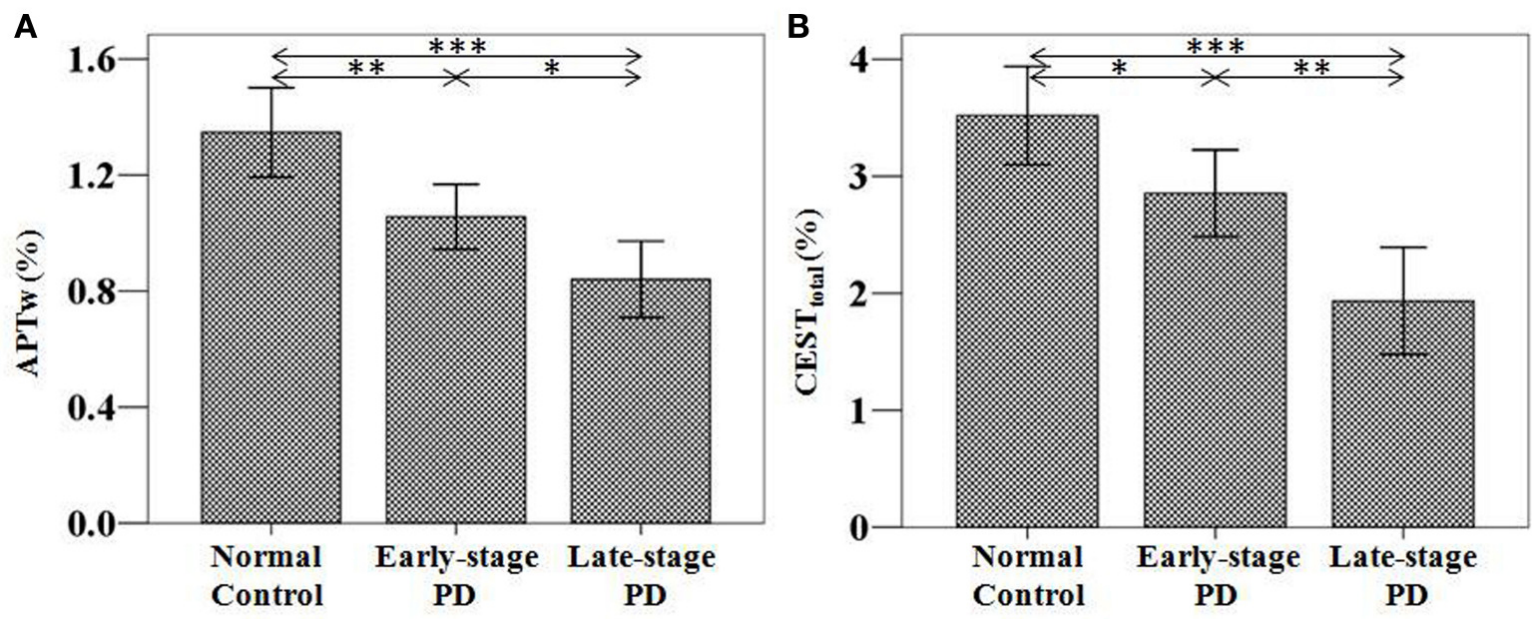
APTw **(A)** and CEST_total_ **(B)** values of the substantia nigra for normal controls (*n* = 24) and PD patients at different stages (grouped by disease duration) (*n* = 32 for early-stage and *n* = 29 for late-stage). The APTw and CEST_total_ values of the substantia nigra for normal controls, early-stage, and late-stage PD patients were (1.35 ± 0.15) and (3.52 ± 0.42)%, (1.06 ± 0.11) and (2.85 ± 0.37)%, (0.84 ± 0.13) and (1.93 ± 0.46)%, respectively. Both showed a gradual decrease with disease progression. ^*^P < 0.05; ^**^P < 0.01; ^***^P < 0.001.

Based on the classification based on disease duration, 46 samples from 32 patients at the early stage were acquired. For the unilateral symptoms group (18 patients), we select one sample on the contralateral substantia nigra of symptoms (18 samples); for the bilateral symptoms group (14 patients), we selected two samples on the bilateral substantia nigra (28 samples). Fifty-five samples from 29 patients at the advanced stage were also acquired (three samples from the three patients with unilateral symptoms; 52 samples for other 26 patients with bilateral symptoms). The results are shown in Figure 5 and Table 2.

**Table 2:** APTw and CEST_total_ values (mean ± 95% CI) of the substantia nigra for normal controls and Parkinson's disease (PD) patients at different stages (classification according to disease duration).

	**Normal (*n* = 24)**	**Early-stage PD (*n* = 32)**	**Late-stage PD (*n* = 29)**	***P*-values**
APTw (%)	1.35 ± 0.15	1.06 ± 0.11	0.84 ± 0.13	**0.003, 0.024**,<**0.001**
CEST_total_(%)	3.52 ± 0.42	2.85 ± 0.37	1.93 ± 0.46	**0.031, 0.002**,<**0.001**

*APTw and CEST_total_ were quantified by % water signal intensity. The three P-values correspond to the results of the comparison between normal controls and early stage PD patients, between early-stage and late-stage PD patients, and between normal controls and late-stage PD patients, respectively. Bold indicates a significant change*.

Both the APTw and CEST_total_ values were able to differentiate PD patients from normal controls, regardless of the PD stage (the early stage or the advanced/late stage). In addition, both APTw and CEST_total_ values of the substantia nigra gradually decreased with disease progression, regardless of which classification (the H&Y scale or disease duration) was used.

**Figure 6:**
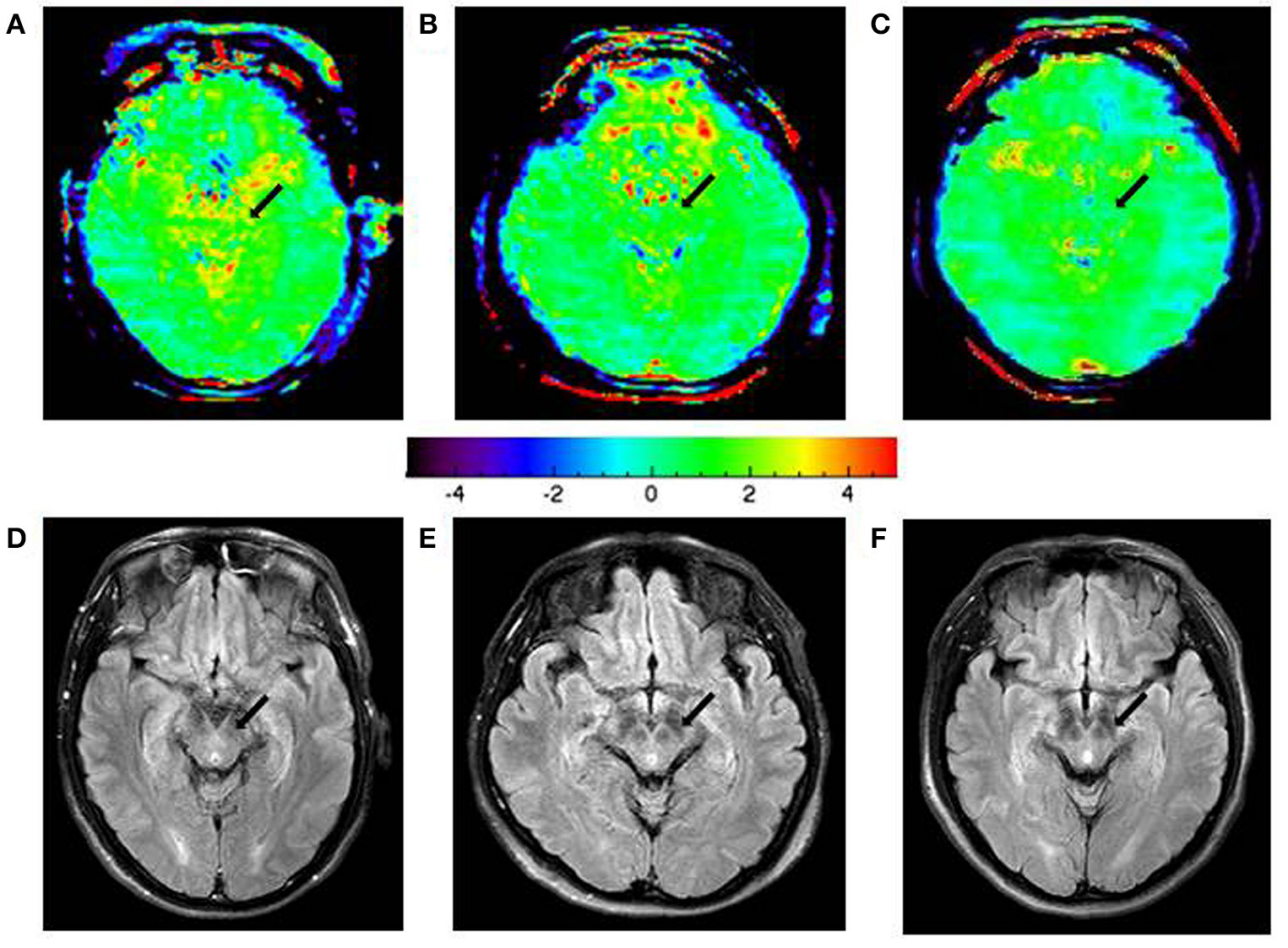
APTw images and FLAIR images of a typical normal control **(A,D)**, an early-stage PD patient **(B,E)**, and an advanced/late-stage PD patient **(C,F)**. The APTw intensities in the substantia nigra (black arrows) were lower in PD patients (green color at the early stage, light blue color at the advanced/late stage) than in the normal controls (scattered yellow color).

Figure 6 shows the typical APTw and FLAIR images of a normal control (male, 54 years old), an early stage PD patient (female, 67 years old, H&Y scale of 2, disease duration of 2 years), and a advanced/late stage PD patient (female, 46 years old, H&Y scale of 3, disease duration of 6 years). On the APTw images, the substantia nigra (black arrows) showed intermediate punctate hyperintensity for the normal control, iso-intensity for the early stage PD patient, and mild hypointensity for the advanced/late stage PD patient. On the FLAIR images, there was no obvious difference in the substantia nigra areas (black arrow) of these three cases.

The APTw and CEST_total_ values of the occipital NAWM are shown in Tables 3, 4. It is due to the presence of the confounding factors relating to $\text{MT}{{\text{R}}^{\prime }}_{\text{asym}}$ that the APTw values in the NAWM area were negative (Zhou et al., 2013a). No significant differences for these two parameters were found between PD patients and normal controls.

**Table 3:** APTw and CEST_total_ values (mean ± 95% CI) of the occipital NAWM for normal controls and Parkinson's disease (PD) patients at different stages (classification according to the H&Y scale).

	**Normal (*n* = 24)**	**Early-stage PD (*n* = 38)**	**Advanced-stage PD (*n* = 23)**	***P*-values**
APTw (%)	−0.12 ± 0.09	−0.11± 0.10	−0.10 ± 0.11	0.838, 0.921, 0.771
CEST_total_(%)	0.95 ± 0.23	0.97 ± 0.21	1.03 ± 0.14	0.891, 0.649, 0.568

*APTw and CEST_total_ were quantified by % water signal intensity. The three P-values correspond to the results of the comparison between normal controls and early stage PD patients, between early-stage and advanced-stage PD patients, and between normal controls and advanced stage PD patients, respectively*.

**Table 4:** APTw and CEST_total_ values (mean ± 95% CI) of the occipital NAWM for normal controls and Parkinson's disease (PD) patients at different stages (classification according to disease duration).

	**Normal (*n* = 24)**	**Early-stage PD (*n* = 32)**	**Late-stage PD (*n* = 29)**	***P*-values**
APTw (%)	−0.12 ± 0.09	−0.11 ± 0.10	−0.11 ± 0.09	0.824, 0.975, 0.791
CEST_total_(%)	0.95 ± 0.23	0.95 ± 0.22	1.03 ± 0.15	0.974, 0.570, 0.543

*APTw and CEST_total_ were quantified by % water signal intensity. The three P-values correspond to the results of the comparison between normal controls and early stage PD patients, between early-stage and late-stage PD patients, and between normal controls and late-stage PD patients, respectively*.

### Differentiating PD at different stages with the CEST/APT signal

We further compared the CEST/APT differences between early-stage PD patients and advanced or late-stage patients. With disease progression (from the early stage to the advanced or late stage), the CEST signal intensities of the substantia nigra significantly decreased, as expected, regardless of which classification was used, which indicated the potential of CEST/APT imaging to differentiate PD patients at different stages.

### CEST/APT signal features for patients with unilateral symptoms

**Figure 7:**
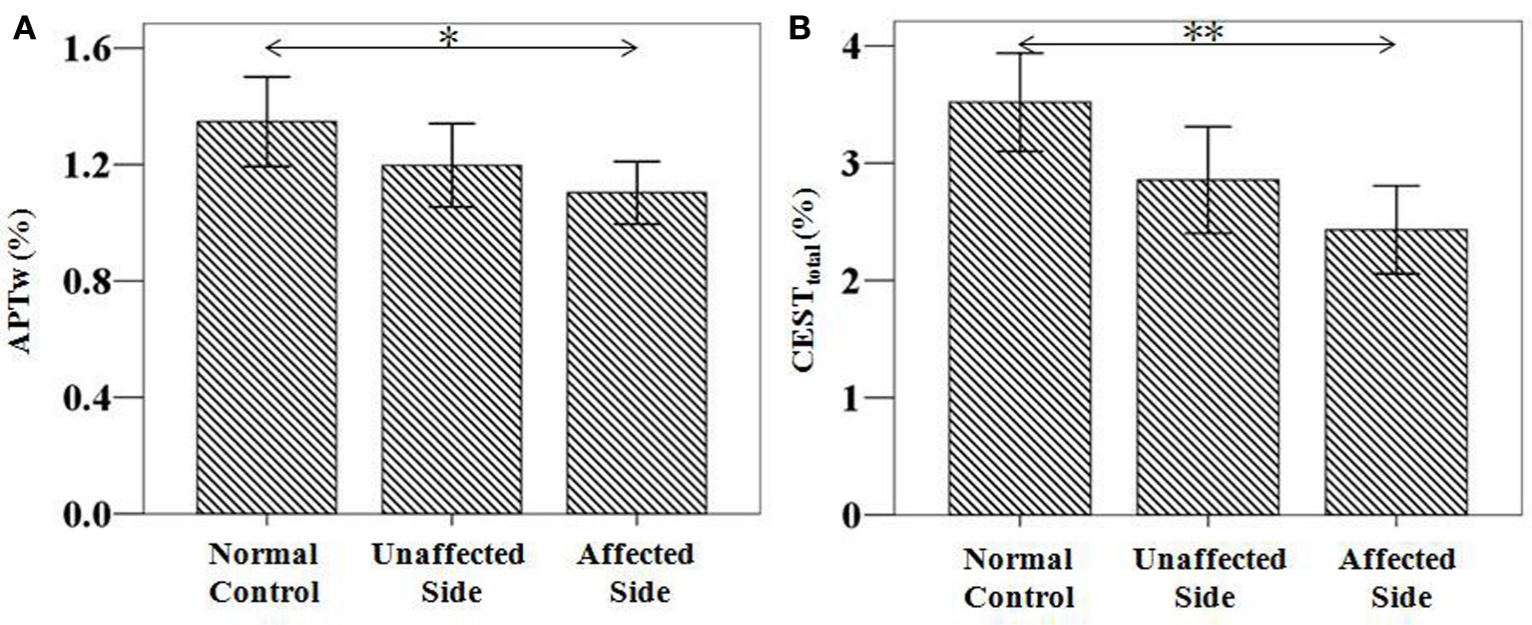
APTw and CEST_total_ values of the substantia nigra for patients with unilateral symptoms (*n* = 21). Both showed a significant decrease in the affected side [APTw: (1.10 ± 0.11)%; *P* = 0.027; CEST_total_: (2.43 ± 0.38)%; *P* = 0.001], compared to normal controls [APTw: (1.35 ± 0.15)%; CEST_total_: (3.52 ± 0.42)%]. ^*^P < 0.05; ^**^P < 0.01.

The CEST/APT imaging intensities of the affected side and unaffected side are compared in Figure 7. Compared to normal controls, both the APTw and CEST_total_ values in the substantia nigra of the affected side were significantly lower [APTw: (1.10 ± 0.11)%; *P* = 0.027; CEST_total_: (2.43 ± 0.38)%; *P* = 0.001]. The APTw and CEST_total_ values in the substantia nigra of the unaffected side seemed to be lower than normal controls, but the differences were not significant [APTw: (1.20 ± 0.15)%; *P* = 0.315; CEST_total_: (2.86 ± 0.46)%; *P* = 0.081]. The APTw and CEST_total_ values in the substantia nigra of the affected side seemed to be lower than unaffected side, but the differences were not significant (APTw: *P* = 0.526; CEST_total_: *P* = 0.303).

### Correlations between the CEST/APT signal and severity evaluation

The severity evaluation of PD is commonly based on multiple scale ratings of different aspects, including the H&Y scale, the UPDRS, disease duration, the MMSE, the HAMD, and the HAMA. The H&Y scale and the UPDRS reflect the overall severity of PD, while the MMSE, the HAMD, and the HAMA mainly reflect the mental status of PD patients. We performed a correlation analysis of the CEST/APT signal with different clinical scales. Both the APTw and CEST_total_ values of the substantia nigra showed significant negative correlations with the H&Y scale, the UPDRS, and disease duration (Table 5). The APTw values also showed a significant negative correlation with UPDRS-III, and the CEST_total_ values also showed significant negative correlations with the UPDRS-II, UPDRS-III, and UPDRS-IV. Neither the APTw nor CEST_total_ values showed significant correlations with the MMSE, the HAMD, or the HAMA.

**Table 5:** Correlation results between the substantia nigra CEST/APT signals and the clinical evaluation.

	**APTw**		**CEST**_**total**_	
	***r***	***P***	***r***	***P***
H&Y scale	−**0.310**	**0.002**	−**0.315**	**0.001**
UPDRS	−**0.334**	**0.001**	−**0.247**	**0.013**
Disease duration	−**0.329**	**0.001**	−**0.315**	**0.001**
UPDRS-I	0.052	0.604	0.059	0.559
UPDRS-II	−0.191	0.056	−**0.219**	**0.028**
UPDRS-III	−**0.399**	<**0.001**	−**0.220**	**0.027**
UPDRS-IV	−0.122	0.223	−**0.217**	**0.029**
UPDRS-V	0.039	0.700	−0.152	0.130
MMSE	−0.114	0.257	0.050	0.618
HAMD	0.038	0.709	−0.158	0.116
HAMA	0.142	0.156	0.097	0.337

*Bold values mean P < 0.05*.

## Discussion

In this study, we investigated the potential of CEST/APT imaging for the diagnosis of PD and the evaluation of disease severity. We quantified the CEST/APT signal intensities of PD patients at different stages and compared the results with normal controls. We classified PD patients into two groups, based on the H&Y scale and based on disease severity, one of which has been used in a previous study (Li et al., 2014), namely, the H&Y stages (early stage, H&Y stages ≤ 2 vs. advanced stage, H&Y stages ≥ 2.5). Our present results revealed the efficiency of CEST/APT signal intensities in the detection of PD, even at the early stage of the disease. This is somewhat different from the previous study, which showed no significant difference between early PD patients and normal controls. Two major factors may explain this difference. The first factor is that we excluded the data from the unaffected side of PD patients with unilateral symptoms, which may have counterbalanced the decrease in CEST/APT signal intensities in PD patients at the early stage, as these decreases may not be as significant as those of the affected side. The comparisons among normal controls with the affected side and the unaffected side of PD patients in our study have clearly confirmed this point, which will be discussed later. The second factor may be the larger sample number in the present study, which may make the differences more obvious and consistent. Notably, we also further classified PD patients based on a different standard—disease duration (early stage, disease duration ≤ 3 year vs. late stage, disease duration > 3 years). This classification is more practical in the clinic, because the H&Y stages require the professional evaluation of neurologists, while the disease duration information can be more easily acquired from the patients. The results showed that, compared with normal controls, PD patients showed significantly lower CEST/APT intensities, regardless of the stage of PD. The results indicated that the clinical use of CEST/APT imaging in the diagnosis of PD would not be influenced by the H&Y stage or the disease duration. Thus, the CEST/APT signals of the substantia nigra may be used as a potential biomarker in the diagnosis of PD. Such results may be ascribed to the substantia nigra dopaminergic cell loss in PD patients, which would unavoidably decrease the CEST-detectable mobile proteins and peptides and other biomolecules in the substantia nigra.

Besides the substantia nigra, we also assessed one more region, the occipital NAWM at the slice of midbrain, which is probably not affected by PD. We did this to examine whether the CEST/APT signal loss is specific for the substantia nigra or the signal changes in other brain regions can also show similar trends. The results (Tables 3, 4) showed that the CEST/APT signals are stable in the NAWM in the different groups.

It is important to note that the significantly reduced CEST_total_ and APTw values were observed in the substantia nigra in the early-stage patients (H&Y stages ≤ 2 or disease duration ≤ 3 year). As mentioned previously, the early diagnosis of PD would play an important part in the management of PD patients, while early diagnosis is still challenging in the clinic, to date. Our results show the capability of CEST/APT as a biomarker in the early diagnosis of PD, which may assist in early intervention for PD patients.

When we compared the CEST signals in the substantia nigra between the normal controls, the affected side of PD, and the unaffected side of PD, we found that both the affected side and unaffected side showed lower CEST signals, particularly the affected side. Our results in the present study reflect the different degree of CEST signal decrease for the affected side and the unaffected side. When the unilateral symptoms progress to bilateral symptoms, the CEST signal intensities decrease aggravatingly. As mentioned previously, the CEST signal decrease in the substantia nigra may be attributable to the dopamine neuron loss. Our results in the present study may reflect the different degree of dopamine neuron loss between the affected and unaffected sides. When the dopamine neuron loss progresses in the unaffected side to a certain point, the unilateral symptoms may progress to bilateral symptoms. Such a result indicated that CEST signals would provide promising information in monitoring the progression of PD, from the preclinical stage to the clinical stage. However, we should notice that no significant differences were found between the affected side and the unaffected side of PD, though the value of affected side seemed to be lower than the unaffected side of PD. This may be attributed to the periodic change of substantia nigra during the PD progression. Such results need to be further confirmed in the future.

Both the APTw and CEST_total_ values in the substantia nigra had significantly negative correlations with the H&Y scale, the UPDRS, and disease duration, which correlated with the progression of PD. The progressively reduced CEST signal may be ascribed to the gradually decreased dopaminergic cell density in the substantia nigra, which is reflected by the worsening condition of PD patients. This may explain why the CEST signal has the potential for the overall severity evaluation of PD. Moreover, the APTw of the substantia nigra showed a significant negative correlation with the UPDRS-III, while the CEST_total_ values showed negative correlations with the UPDRS-II, the UPDRS-III, and the UPDRS-IV. It is known that the UPDRS-II, the UPDRS-III, and the UPDRS-IV measure the activities of daily living, motor complications, and treatment complications, respectively. Therefore, the negative correlations between the CEST signal and the UPDRS-II, the UPDRS-III, and the UPDRS-IV may indicate the potential of CEST for the further evaluation of the sub-score of the UPDRS and provide more information for disease assessment in PD. No significant correlations were found for the CEST signals with the mental status (the MMSE, the HAMD, and the HAMA). This could be explained by the fact that the mental status of PD patients may be influenced by multiple factors, in addition to the substantia nigra changes.

The results for CEST_total_ and APTw seemed very similar, but some different points were found between these two CEST metrics. For example, APTw signal differences between the early-stage PD patients and the normal controls seemed to be more obvious than the CEST_total_, indicating that “protein-based” APTw at 3.5 ppm may be more sensitive in detecting early-stage PD patients than the CEST_total_, which may derive from multiple chemical sources (mobile proteins and peptides, brain metabolites, etc., quantified by the integral of the MTR_asym_ spectrum in the range of 0 to 4 ppm). In addition, CEST_total_ may be more sensitive in differentiating early-stage patients from advanced or late-stage patients than APTw. There were also some differences for the correlation results. APTw values had a negative correlation with the UPDRS-III, while CEST_total_ had negative correlations not only with the UPDRS-III, but also with the UPDRS-II and the UPDRS-IV. These results may be attributable to the different underlying meanings for these two metrics.

The decreased MTR_asym_ spectrum of the substantia nigra for PD patients appeared at a wide offset range (Figure 3), suggesting that the APTw and CEST_total_ decreases could be attributed to the loss of many kinds of water-exchanging chemicals (mobile proteins and peptides, brain metabolites, etc.) caused by the loss of dopaminergic neurons. The depletion of some chemicals with fast-exchanging protons, such as dopamine and glutamate, may also reduce the whole MTR_asym_ spectrum as a result of the wide saturation width. However, the detection of the chemicals with fast-exchanging protons is not favorable at 3 Tesla. Therefore, the loss of dopaminergic neurons would be the more likely explanation for a decline in the whole MTR_asym_ spectrum. The exact mechanisms for these changes in APTw and CEST_total_ intensities still need to be further studied in the future.

There were some limitations to this study. First, we used a single-slice CEST/APT imaging acquisition protocol, but we attempted to include the maximum slice of the substantia nigra when selecting the scan slice. In a future study, we will increase the CEST/APT sequence coverage by using a 3D imaging acquisition sequence, which has been reported in previous studies (Zhao et al., 2013; Zhou et al., 2013b), so that the whole substantia nigra can be acquired. Also, we will be able to assess the other brain regions besides the slice of midbrain. Second, the spatial resolution of CEST/APT imaging needs improvement in future studies, so that we can describe the substantia nigra regions more precisely.

In conclusion, CEST/APT imaging is a novel MRI technique with which to detect PD patients at any disease stage. The significant APTw signal difference in the substantia nigra between early-stage PD patients and normal controls indicated the capability of CEST/APT for the early diagnosis of PD, which would make possible early intervention in PD. The correlations between the CEST signals and clinical evaluations indicated that the CEST signals can reflect disease severity. The CEST signal thus has the potential to serve as an imaging biomarker for assessing the progression of PD.

## Author contributions

CL, MC, and JZhou designed research; CL, RW, HC, WS, SL, GS, and JZhang conducted research; XZ, BL, and SZ analyzed data; CL, XZ, and JZhou wrote the manuscript. All authors read and approved the manuscript.

### Conflict of interest statement

The authors declare that the research was conducted in the absence of any commercial or financial relationships that could be construed as a potential conflict of interest.
